# Successful Conservative Management of a Blowout Fracture With Diplopia Using Self-Performed Manual Rehabilitation: A Case Report

**DOI:** 10.7759/cureus.88380

**Published:** 2025-07-20

**Authors:** Teruyuki Niimi, Ken Kitagawa, Chisato Sakuma, Hiroo Furukawa, Nagato Natsume

**Affiliations:** 1 Division of Research and Treatment for Oral and Maxillofacial Congenital Anomalies, School of Dentistry, Aichi Gakuin University, Nagoya, JPN; 2 Cleft Lip and Palate Center, Aichi Gakuin University Dental Hospital, Nagoya, JPN

**Keywords:** blowout fracture, conservative management, diplopia, facial trauma, manual rehabilitation

## Abstract

Early surgical intervention is generally recommended for blowout fractures presenting with diplopia or restricted ocular motility. We report a case of a blowout fracture in which diplopia and mild upward gaze limitation were observed immediately after the injury, but a favorable outcome was achieved through conservative management involving self-administered manual rehabilitation.

The patient was a 59-year-old man who sustained facial trauma due to a fall. CT imaging revealed a blowout fracture with herniation of orbital contents into the maxillary sinus. Physical examination showed periorbital swelling, tenderness, and diplopia during upward gaze in the left eye, while visual acuity and other ocular movements remained normal. Although surgical repair was initially considered, the patient noticed that manually elevating the globe through the lower eyelid improved his diplopia and opted for conservative treatment. He performed daily manual globe elevation exercises. Within one month, the diplopia had almost completely resolved. Mild, intermittent recurrence occurred two months later but resolved promptly upon resuming the exercises. At the two-year follow-up, the patient had no symptoms of ocular motility disturbance or diplopia.

This case suggests that self-performed manual globe elevation may serve as an effective conservative treatment option in selected cases of blowout fracture where surgical intervention is not feasible. Although further studies are warranted, this simple and noninvasive method may be useful in the management of post-traumatic diplopia.

## Introduction

The orbit is composed of the maxilla, palatine, zygomatic, sphenoid, lacrimal, ethmoid, and frontal bones, and it houses the eyeball, optic nerve, extraocular muscles, and lacrimal gland, as well as associated nerves, blood vessels, and fat. While the entrance of the orbit (the area surrounding the eye) is relatively strong, the medial and inferior walls located deeper within the orbit are thin and fragile. Therefore, trauma or other causes can lead to fractures of the thin orbital walls, and when part of the orbital contents herniates into the maxillary sinus due to a fracture of the orbital floor, it is referred to as a "blowout fracture."

Blowout fractures are often treated conservatively when there are no aesthetic or functional abnormalities [1]. However, when ocular displacement or diplopia is present, early surgical intervention, consisting of reduction of herniated orbital contents and reconstruction of the orbital floor, is generally recommended [2-4]. Nevertheless, surgical treatment may not be feasible due to the patient's general condition or personal preference [5]. We report a rare case of a blowout fracture in which mild upward gaze limitation and diplopia were observed immediately after the injury, but favorable outcomes were achieved through manual rehabilitation alone.

## Case presentation

A 59-year-old male sustained facial trauma after a fall while walking. He presented with epistaxis but no loss of consciousness. He was transported to an emergency hospital and diagnosed with a blowout fracture and a sprain of the left wrist. He received antibiotics and analgesics. On the following day, further evaluation was conducted at our hospital. Physical examination revealed swelling and tenderness around the left eye, as well as diplopia. Due to the diplopia, the patient was unable to walk with both eyes open. Although upward gaze limitation of the left eye was observed, other ocular movements were possible, and visual acuity was preserved.

**Figure 1:**
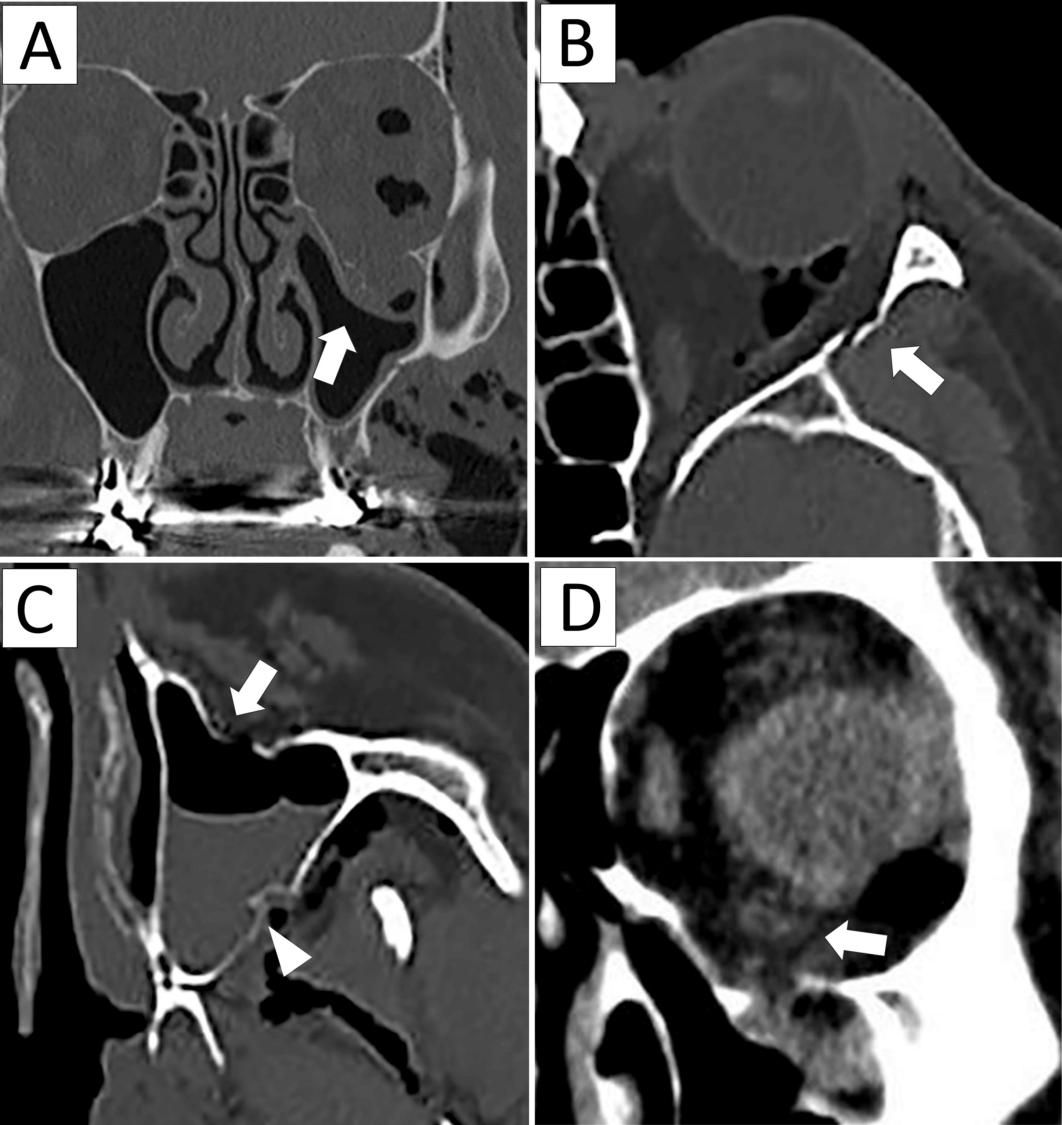
CT images A. Herniation of orbital contents into the maxillary sinus (arrow). B. Fracture of the lateral orbital wall (arrow). C. Fractures of the anterior wall (arrow) and lateral wall (arrowhead) of the maxillary sinus, with hematoma in the sinus. D. The inferior rectus muscle (arrow) remained within the orbit and did not herniate into the maxillary sinus.

CT imaging confirmed a blowout fracture of the orbital floor with herniation of orbital contents into the maxillary sinus. The inferior rectus muscle remained within the orbit and did not herniate into the maxillary sinus. Additional fractures of the lateral orbital wall, anterior wall, and lateral wall of the maxillary sinus were noted, with hematoma accumulation in the sinus (Figure 1).

There was no zygomatic arch fracture, and occlusion remained normal. Extensive subcutaneous emphysema from the forehead to the submandibular area was observed, along with hypoesthesia in the midface region. Although there was wrist pain, no fracture was identified. A diagnosis of blowout fracture was made.

**Figure 2:**
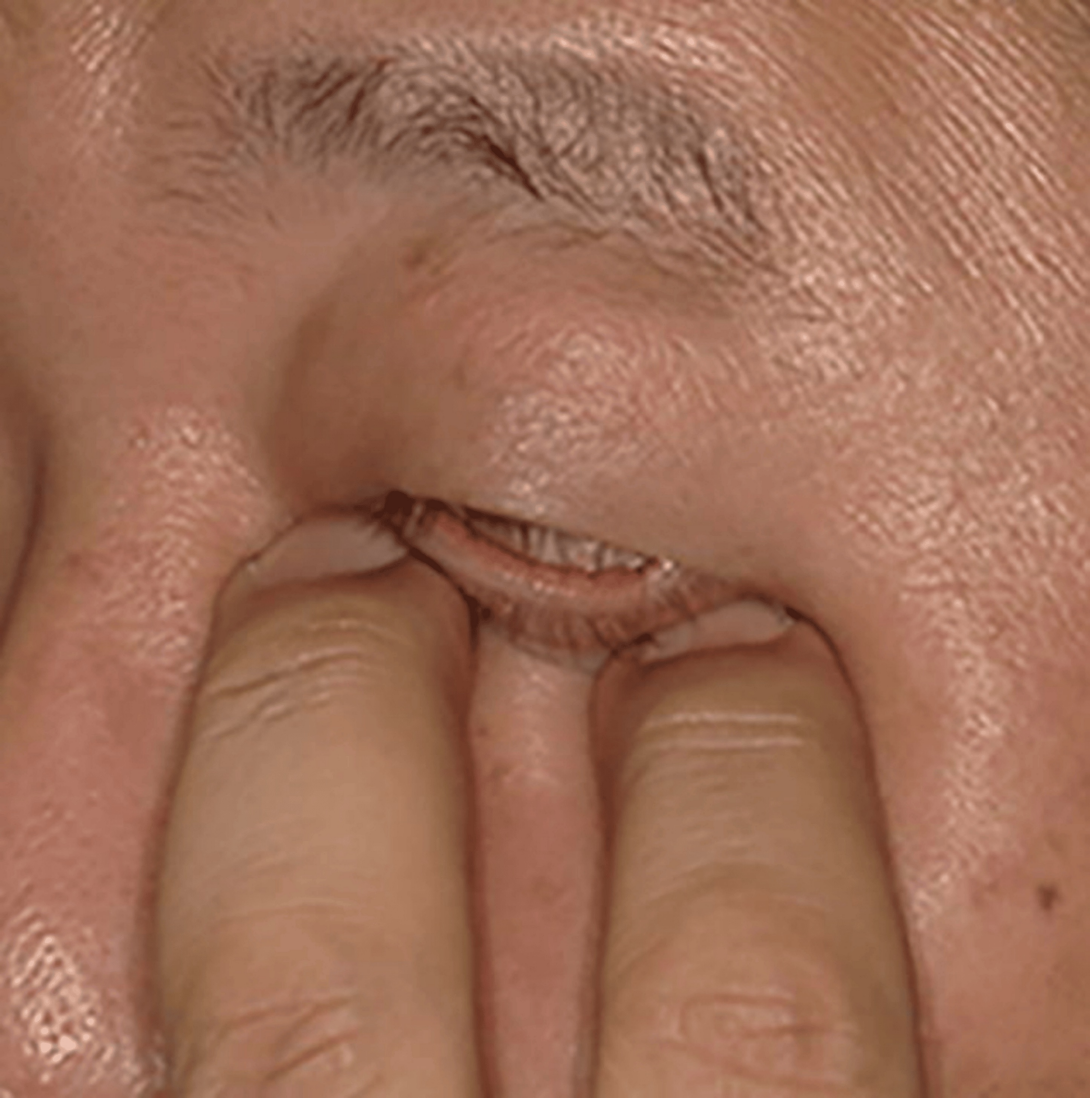
Self-rehabilitation. Manual upward elevation of the globe through the lower eyelid using the patient's fingers.

Surgical repair was considered necessary. However, since the patient noticed improvement of diplopia when manually elevating his left globe upward through the lower eyelid, he opted against surgery. It was agreed to proceed with conservative treatment and reassess the need for surgery if diplopia persisted. The patient performed manual upward globe elevation daily for several minutes. This rehabilitation led to gradual improvement in diplopia (Figure 2).

**Figure 3:**
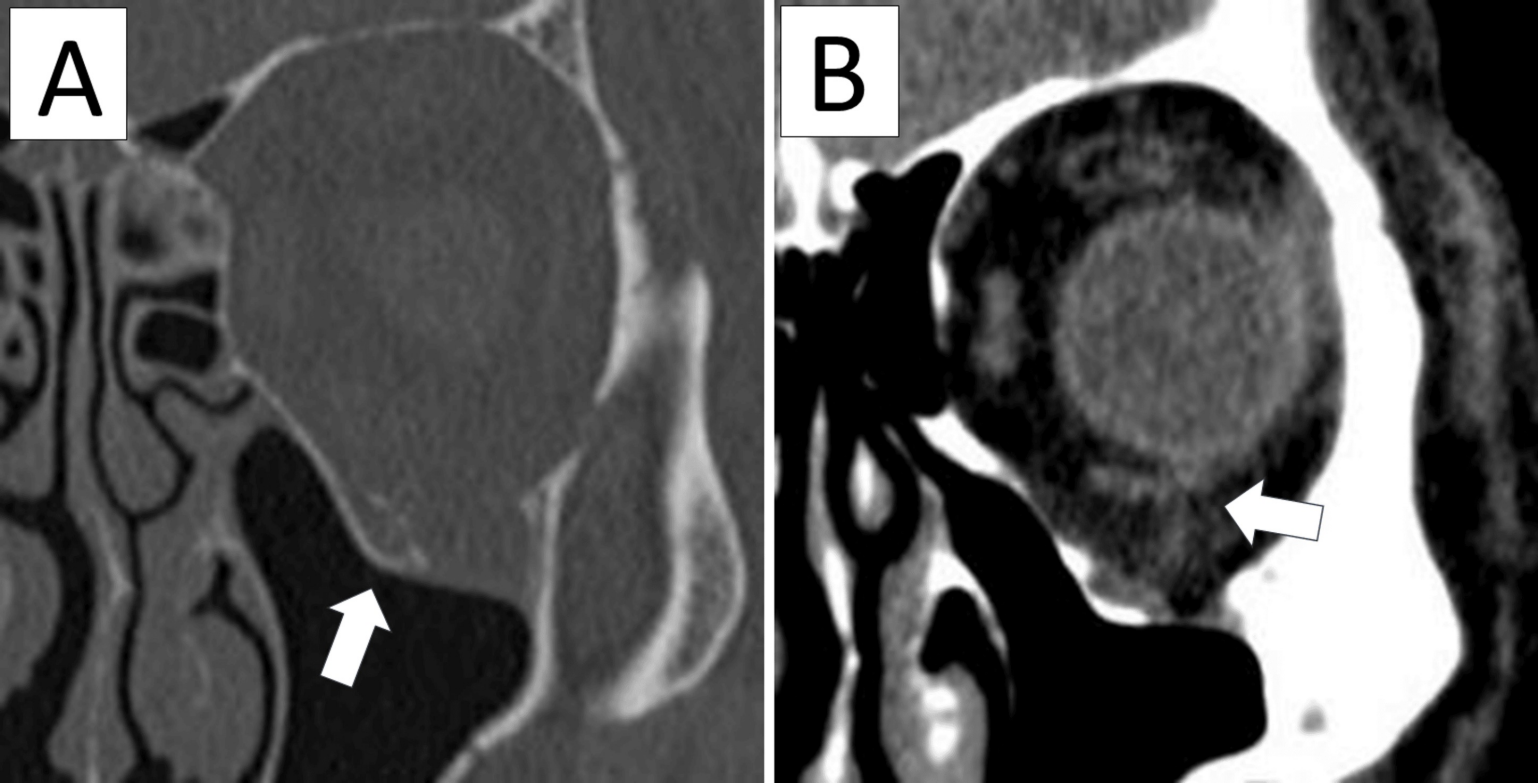
CT at five months post-injury The orbital floor bone (arrow) has mostly healed in a displaced position. The inferior rectus muscle (arrow) has returned to its normal location.

Cheek swelling resolved within 10 days, pain subsided within two weeks, and diplopia nearly disappeared within one month, allowing the patient to discontinue rehabilitation. Around two months later, the patient occasionally experienced mild diplopia again, which promptly resolved with the same manual maneuver. The patient was seen daily for the first week and then once a month for two years. A CT scan performed at five months post-injury showed that the orbital floor bone had mostly healed in a deformed state, with no abnormal positioning of the inferior rectus muscle (Figure 3). At the two-year follow-up, although no detailed ophthalmologic examinations were performed, there were no clinically observed ocular motility disturbances or diplopia, indicating a favorable clinical course.

## Discussion

Blowout fractures are caused by trauma such as traffic accidents, sports injuries, physical assault, or falls [6]. Symptoms include globe displacement, periorbital swelling, pain, ecchymosis, restricted ocular motility, and diplopia [7]. CT imaging typically shows herniation of orbital contents into the maxillary sinus [8]. Mild cases may be managed conservatively with anti-inflammatory treatment alone. However, surgical repair is generally recommended in cases with ocular motility impairment or diplopia to reposition the orbital contents and reconstruct the orbital floor [2,3,9-11].

According to Burnstine's review, emergency surgical intervention is indicated in cases involving a non-resolving oculocardiac reflex, “white-eyed” blowout fractures, and early onset of enophthalmos or hypoglobus [3]. In addition, surgery within two weeks is recommended for symptomatic diplopia with positive forced duction tests and CT evidence of orbital soft tissue entrapment, or in the presence of large blowout fractures that may cause delayed enophthalmos or hypoglobus [3,4]. Pandya et al. suggested emergent surgery for tissue entrapment, and elective intervention for persistent diplopia, enophthalmos exceeding 2 mm, or fractures involving more than 50% of the orbital floor [5]. However, the timing, indications, and surgical approach remain subjects of debate. Furthermore, when surgery is not an option due to systemic conditions or patient refusal, the optimal conservative management to mitigate sequelae such as diplopia remains unclear.

In this case, ocular motility impairment was mild, and visual acuity was normal. Although diplopia was present, the patient could alleviate it by manually elevating the eye upward from the lower eyelid. Respecting the patient’s preference, early surgery was deferred. The patient continued self-administered manual globe elevation rehabilitation daily, which coincided with almost complete resolution of diplopia within one month. After discontinuing the exercises, diplopia occasionally recurred but resolved with repeated rehabilitation.

This report describes the improvement of diplopia through self-administered rehabilitation in a case of mild blowout fracture. To our knowledge, while many reports discuss surgical treatment of blowout fractures, there are no previous reports describing conservative management using manual globe elevation as rehabilitation. After providing a thorough explanation that surgery would be performed immediately if diplopia did not improve, the patient undertook self-rehabilitation. Although this is a single case and does not establish the efficacy of the method, it is a simple and noninvasive approach. It may be a viable conservative treatment option when surgery is not feasible.

## Conclusions

This case highlights the potential of conservative management in select blowout fracture patients presenting with mild diplopia and ocular motility limitation. Despite imaging evidence of herniated orbital contents, our patient achieved near-complete resolution of symptoms through daily manual globe elevation, without surgical intervention. To our knowledge, this is the first report describing such a rehabilitation method for diplopia associated with blowout fractures. Although further studies are needed to assess its efficacy, manual globe elevation may serve as a simple, noninvasive treatment option when surgery is not feasible due to medical or personal factors.
